# Early Cortisol and Inflammatory Responses to Parental Cancer and Their Impact on Functional Impairment in Youth

**DOI:** 10.3390/jcm10040576

**Published:** 2021-02-04

**Authors:** Benjamin Hayes, Jacob Brent, Yongqi Zhong, Shervin Bazmi, Giovanna Porta, Dana H. Bovbjerg, Ahmad Tarhini, John M. Kirkwood, David A. Brent, Anna Marsland, Nadine M. Melhem

**Affiliations:** 1 University of Pittsburgh School of Medicine, 3550 Terrace St, Pittsburgh, PA 15213, USA; BJH89@pitt.edu; 2 Department of Psychiatry, University of Pittsburgh School of Medicine, 3550 Terrace St, Pittsburgh, PA 15213, USA; brentj@upmc.edu (J.B.); brentda@upmc.edu (D.A.B.); 3 Department of Epidemiology, University of Pittsburgh Graduate School of Public Health, 130 De Soto St, Pittsburgh, PA 15261, USA; yoz21@pitt.edu; 4 Department of Psychology, University of Pittsburgh, 210 South Bouquet Street, Pittsburgh, PA 15260, USA; bazmis2@pitt.edu (S.B.); marsland@pitt.edu (A.M.); 5 University of Pittsburgh Medical Center (UPMC), 100 N Bellefield Ave, Pittsburgh, PA 15213, USA; portgx@upmc.edu; 6 UPMC Hillman Cancer Center, 5150 Centre Ave. Pittsburgh, PA 15232, USA; bovbjergdh@upmc.edu (D.H.B.); Ahmad.Tarhini@moffitt.org (A.T.); kirkwoodjm@upmc.edu (J.M.K.)

**Keywords:** chronic stress, parental cancer, functional impairment, cortisol, inflammation, cancer and oncology

## Abstract

Purpose: Chronic stress is associated with increased risk for maladaptive psychological responses during childhood, adolescence, and young adulthood. Adults exposed to chronic stress during childhood exhibit dysregulation of hypothalamic-pituitary-adrenal (HPA) axis activity and inflammation. There are no studies examining the impact of stress on biological stress responses and functional impairment in adolescents and young adults early after the onset of a stressor. Methods: The sample consisted of 59 offspring, aged 11–25 years, 33 of parents diagnosed with cancer and 26 controls from families with no cancer or severe chronic illness in parents or siblings. Cancer patients and their families were recruited within an average of 62 days (SD = 35.9) and followed at 6 and 9 months later. Functional impairment was assessed and hair cortisol concentrations (HCC), salivary cortisol, and inflammatory markers were measured. Mixed regression analyses were conducted. Results: The stress group showed higher functional impairment (β = −5.5, 95% CI (−10.4, −0.06), *p* = 0.03, d= −0.40) and HCC (β = 10.5, 95% CI (−5.5, −0.50), *p* < 0.001, d = 1.43). However, HCC were reduced over time in the stress group (β= −0.3, 95% CI (−0.04, −0.01), *p* < 0.001, d = −1.08). Higher total cortisol output was associated with increased functional impairment over time (β = −3.0, 95% CI (−5.5, −0.5), *p* = 0.02, d = −0.60). Conclusions: Parental cancer is associated with early increase in cortisol, which was associated with increased functional impairment in offspring. Clinicians need to assess and monitor psychiatric symptoms and functioning in these offspring early on following parental cancer diagnosis.

## 1. Introduction

Cancer is the second leading cause of death in the United States, with an estimated economic impact at over a hundred billion dollars annually for treatment and morbidity-related expenses including disability [1] and loss of productivity [2]. An estimated three million children live with a parent who has been diagnosed with cancer in the United States [3]. A cancer diagnosis is a stressful life event for patients and their families. However, its impact on their offspring is understudied, even though facing the possibility of losing a parent to cancer and witnessing the course of cancer treatment is a major stressor for offspring.

Epidemiological studies have shown increased rates of depression, anxiety, and other clinical symptomatology among children of parents diagnosed with cancer [4]. Similar findings of increased psychiatric morbidity [5,6] as well as overall mortality [7] are reported in those who have experienced childhood parental death. Studies previously reported an increased risk for psychiatric disorders and functional impairment to extend several years following the parent’s death, potentially into adulthood [7,8].

The hypothalamic-pituitary-adrenal (HPA) axis is activated in response to psychological stress, resulting in the secretion of cortisol. Cortisol has profound implications across a wide variety of endocrine and immunologic functions, particularly in the regulation of systemic inflammation. Studies have shown that adults exposed to chronic stress during childhood exhibit increased peripheral levels of C-Reactive Protein (CRP) and proinflammatory cytokines such as Interleukin-6 (IL-6), which could put them at increased risk for psychopathology [9]. It has been hypothesized that chronic stress may lead to dysregulation in the HPA axis through reduced glucocorticoid receptor (GR) sensitivity in response to the chronic production of cortisol, resulting in the downstream activation of inflammatory pathways [10]. Studies also suggest a long-lasting reprogramming of endocrine and inflammatory phenotypes in adults exposed to early life adversity [11]. However, it is not clear whether such reprogramming is observed in youth early on following exposure to a major life stressor. There is a paucity of longitudinal research examining the impact of major stressors on the HPA axis and inflammatory markers and whether these could serve as potential markers of risk for early onset of psychopathology. More specifically, there is no research examining parental cancer as a model of stress in adolescents and young adults, which will allow us to examine changes in biological markers early on after the onset of the stressor, i.e., parental cancer diagnosis and whether these changes predict the increased risk for psychopathology in this population.

The purpose of this research is to assess the impact of parental cancer on cortisol and inflammatory responses and their relationship to functional impairment in offspring of parents recently diagnosed with cancer. We hypothesize that offspring of parents with cancer will show increased cortisol early on following diagnosis; there will be a downregulation of cortisol responses over time and increased inflammation; and these will be associated with increased functional impairment in offspring.

## 2. Methods

### 2.1. Sample

The sample consisted of 59 offspring from 33 families, aged 11–25 years: 33 from 17 families of parents recently diagnosed with cancer (designated the “stress” group) and 26 controls from 16 families with no cancer or severe chronic illness in parents or siblings. Cancer patients and their families were recruited from the University of Pittsburgh Hillman Cancer Institute within an average of 62 days (standard deviation (SD) = 35.9, range = 18–162 days) from parental cancer diagnosis. Offspring were included only if they had knowledge of their parent’s cancer diagnosis. Control offspring were recruited through the University of Pittsburgh Clinical and Translational Science Institute Research Participant Registry, which enrolls patients at points of routine clinical care at the University of Pittsburgh Medical Center (UPMC), through MyChart, and at community outreach events. Exclusion criteria included Cushing’s and Addison’s diseases, chronic inflammatory conditions, and use of oral or intravenous corticosteroids in the prior year. For all offspring, interviews and self-reported questionnaires were conducted at intake and at two follow-ups (6- and 9-months post-intake); biological measures were only assessed at intake and at the first follow-up (6-months post-intake). Informed consent and assent were obtained as required by the University of Pittsburgh Institutional Review Board, which reviewed and approved this study (STUDY 19080109). The sample was almost equally distributed by sex (58.6% female) with a mean age of 17 years (SD = 3.4, range = 12–25). The majority of the sample was Caucasian (98.3%) not of Hispanic origin. The parent in the stress group was diagnosed with melanoma or other skin cancers in 96.9% of families; one parent had lung cancer (3.1%). Follow-up rate throughout the study was 72.4% for the overall sample; however, the stress group showed significantly lower follow-up rates compared to the controls (53.1% vs. 96.2%, Fisher’s Exact Test or FET, d = −1.7). Offspring who completed the study were similar to those who did not in terms of demographics, prior psychiatric disorders and stressors, psychiatric symptoms, functional impairment, and most biological measures at intake.

### 2.2. Assessments

Lifetime history and current psychiatric disorders were assessed during the interview using the Family History Research Diagnostic Criteria [12] (FH-RDC) for DSM-IV disorders asking about the offspring. From the FH-RDC, a binary composite variable, “History of Psychiatric Disorders”, was created which coded all offspring for the presence and absence of any clinical diagnosis prior to enrollment. Functional impairment at home, school, and with peers was rated by the clinical interviewer using the Children’s Global Assessment Scale (CGAS) [13], where a lower score reflects greater impairment. We also assessed perceived stress using the Perceived Stress Scale [14]; self-reported anxiety symptoms using the Screen for Child Anxiety Related Emotional Disorders [15]; self-reported depression symptoms using the Child Depression Inventory [16]; and post-traumatic stress disorder (PTSD) symptoms using the PTSD Symptom Scale Interview [17]. Self-reported questionnaires assessing risk factors prior to their parent’s cancer diagnosis were also administered, including the assessment of abuse and neglect using the Childhood Trauma Questionnaire [18]; negative life experiences using the Life Events Checklist [19]; and sleep quality using the Pittsburgh Sleep Quality Index [20]. We also measured height and weight and computed body mass index (BMI). Socioeconomic status (SES) was estimated using the Hollingshead Index of Socioeconomic Status [21].

### 2.3. Biological Measures

Hair cortisol concentrations (HCC). Hair samples were collected to measure HCC at intake and the 6-month follow-up. Hair grows ~1-cm segment per month and, as such, the 3-cm segments closest to the scalp would reflect HCC for the past three months [22]. Hair samples were collected and the number of cm segments closest to the scalp to be assayed for HCC at intake was chosen in relation to the time of parental cancer diagnosis in order to reflect cortisol concentrations since parental diagnosis. For the controls at intake, and for both the cancer and controls at the first follow-up, the 3-cm segment closest to the scalp was assayed, reflecting HCC for the 3 months prior to assessment. HCC was quantified in pg/mg using highly sensitive enzyme immunoassay following methods described by Laudenslager et al. [23] All samples were run in duplicate, with inter- and intraassay coefficients of variations (CV) of less than 5%.

Salivary cortisol. We collected salivary samples 5 times a day over the course of two consecutive days, following the MacArthur research network’s recommendations, at pre-specified timepoints: at awakening, 45-min post-awakening and prior to toothbrushing, food intake, or any significant physical activity, eight- and twelve-hours post-awakening, and prior to sleeping. Participants completed diaries on the days of collection inquiring about patterns of waking, sleeping, eating, exercising, smoking, and alcohol consumption, as well as recording times of salivary sampling. All samples were run in duplicate, with coefficient variations of less than 5%. Cortisol awakening response (CAR) and total diurnal cortisol using the area under the curve (AUC_cort_) were analyzed.

C-Reactive Protein (CRP). Blood draws were rescheduled if the participant reported symptoms of acute illness, or had antibiotics, or received a vaccination within the past two weeks, or anti-inflammatories were taken in the past 12 h. Concentrations (mg/dL) were determined using a highly sensitive CRPH reagent(Beckman Coulter, Indianapolis, IN, USA) on clinical chemistry analyzer AU 5800 (Beckman Coulter, Indianapolis, IN, USA).

Stimulated production of IL-6 and glucocorticoid receptor (GR) sensitivity. Whole blood was incubated with increasing cortisol concentrations in the presence of lipopolysaccharide for 18 h. Final cortisol concentrations were 276, 27.6, 2.76, 0.276, 0.0276, and 0 nmol/L. Final concentration of LPS was 2.5 ng/mL. Following incubation, supernatants were harvested and stored at −80 °C. Levels of IL-6 (pg/mL) in the supernatants were assessed using an enzyme-linked immunosorbent assay (BD, Cat # 555220). IL-6 production in the 0 cortisol condition provides stimulated production of IL-6. Glucocorticoid sensitivity was assessed as area under the curve (AUC) calculated by subtracting the unstimulated control from all values (including the stimulated control), and using the trapezoidal method, with zero as ground [24]. Larger AUC corresponds to greater IL-6 or increased glucocorticoid resistance.

### 2.4. Statistical Analysis

Chi-square and t-tests were performed to compare the cancer and control groups in terms of demographics, clinical characteristics, and biological measures at intake. Normality assumptions were examined for biological variables and a logarithmic transformation was applied to non-normalized distributions (CRP and AUC_cort_). We also compared the cancer and control groups in terms of self-reported symptoms and biological markers using linear regression, controlling for sex and age, and BMI for the biological markers. Next, mixed effects regression analyses were used to examine changes over time in functional impairment, including main effects of group, time (measured as days from diagnosis for the stress group in order to standardize across participants’ staggered intake and follow-up measurements), and group by time interaction (Model 1). This regression model was repeated controlling for sex and age (Model 2). Since our participants were clustered within families, we estimated the variance–covariance matrix with correlated or clustered observations and computed clustered robust standard errors. Similar analyses were conducted for changes in biological measures over time (i.e., CRP, AUC_cort_, CAR, and HCC) and additionally controlling for BMI in Model 2. To examine whether changes in biological measures over time predict functional impairment, mixed models were conducted with functional impairment as the dependent variable and including group, time, group by time interaction, biological measure (one at a time), and biological measure by time interaction. We used multiple imputation by chained equations technique for missing data [25]. Similar results were obtained using the original and imputed datasets. We report results from the imputed dataset. Given our relatively small sample size, we report effect sizes (Cohen’s d).

## 3. Results

Sample characteristics. The cancer and control groups were similar in terms of age, SES, and BMI; however, offspring in the stress group were more likely to be female compared to controls (78.1% vs. 34.6%, respectively, χ^2^(1) = 11.2, *p* = 0.001, d = 0.98) (Table 1). The two groups were similar in terms of prior history of any psychiatric disorders (*p* = 0.38), childhood abuse and neglect (*p* = 0.64), and negative life experiences (*p* = 0.81) prior to cancer diagnosis.

Symptomatology at intake. Offspring in the stress group showed lower scores on the CGAS at intake, which represents greater functional impairment compared to the controls (82.2 ± 10.5 vs. 87.8 ± 7.9, respectively, t = −2.1, *p* = 0.04, d = −0.60) (Table 1). Similarly, offspring in the stress group reported higher scores on depression symptoms; anxiety symptoms; and higher perceived stress compared to controls. The two groups were not significantly different in terms of PTSD and sleep problems, although effect sizes (ES) were 0.39 and 0.26, respectively, which are small ES (Table 1). When controlling for sex and age, only depression (β = 5.7, 95% CI (1.4, 9.9), *p* = 0.01, d = 0.50) and perceived stress (β = 7.8, 95% CI (1.4, 14.3), *p* = 0.02, d = 0.48) remained significantly different between groups (Table 2).

**Figure 1:**
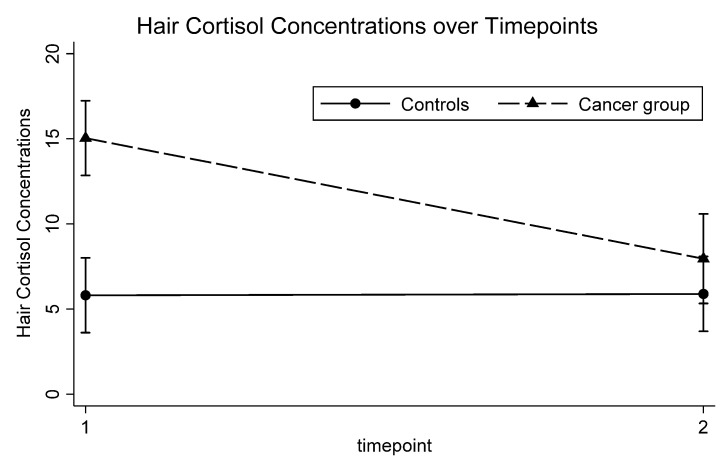
Mean HCC concentrations (pg/mg) over time between cancer and control groups.

Biological measures at intake. HCC was significantly increased in the stress group at intake compared to controls (Table 1, Figure 1). There were no significant differences in CAR although there was a medium effect size. CRP, AUC_cort_, GR sensitivity, and stimulated production of IL-6 were not significantly different between the two groups. Similar results were obtained when controlling for sex, age, and BMI, where HCC was the only measure significantly increased in the stress group (β = 9.2, 95% CI (4.3, 14.1), *p* = 0.001, d = 1.47) (Table 2). Within the stress group, there was no relationship between HCC and time from diagnosis (r = −0.11, *p* = 0.522).

Changes in functional impairment over time. Using mixed regression models, offspring in the stress group showed greater functional impairment compared to controls (β = −5.5, 95% CI (−10.4, −0.6), *p* = 0.03, d = −0.40). There were no changes over time in functional impairment and no group by time interaction (Table 3). Controlling for sex and age, group differences in functional impairment were reduced and did not reach statistical significance (β = −4.7, 95% CI (−9.8, 0.4), *p* = 0.07, d = −0.31).

Changes in biological measures over time. The stress group showed significantly higher HCC (β= 10.8, 95% CI (7.1, 14.4), *p* < 0.001, d = 1.62) compared to controls across timepoints (Table 3). There was also a significant group by time interaction (β= −0.03, 95% CI (−0.04, −0.01), *p* < 0.001, d = −1.14), with the stress group showing reduced HCC over time (Figure 1). There were no significant main effects for group and time and no significant group by time interactions for each of CRP, AUC_cort_, CAR, GR sensitivity, and stimulated production of IL-6. Similar results were obtained when controlling for sex, age, and BMI.

Relationships between functional impairment and biological measures over time. There were no significant main effects for CRP, CAR, HCC, GR sensitivity, and stimulated production of IL-6 on functional impairment even after controlling for group, time, and group by time interaction (Table 4). However, higher total cortisol output or AUC_cort_ was significantly associated with increased functional impairment. In this model, the stress group continued to show significantly increased functional impairment but there was no group by time interaction. Similar findings were obtained when controlling for sex and age.

## 4. Discussion

Offspring of parents diagnosed with cancer showed higher functional impairment within 2 months of parental diagnosis compared to controls, which continued throughout the year following diagnosis. They also showed increased cortisol, as measured by hair cortisol concentrations, early on following diagnosis and at the first follow-up. Our results also show decreased HCC over time in the stress group. Finally, higher total diurnal cortisol (AUC_cort_) was associated with increased functional impairment.

To our knowledge, this is the first study to examine the biological and psychological stress responses in children of parents diagnosed with cancer within ~2 months of diagnosis. Offspring of parents with cancer were not different compared to controls in terms of SES, prior history of any psychiatric disorders, childhood history of abuse and neglect, and negative life events prior to parental diagnosis, yet they showed increased levels of impairment, making this population ideal to study the unfolding of stress responses. Our study has several limitations, including the small sample size. Our study was powered to detect large effect sizes in the order of 0.739 or more, with 80% power and α level of 0.05. This was a feasibility study to examine whether we can recruit children and families undergoing a major stressor such as parental cancer. Another limitation is that our offspring’s age range was wide, comprising several distinct developmental periods including prepubertal and pubertal stages, which may impact some of our findings, especially those related to biological measures. Furthermore, the stress group consisted of more females compared to the control group. We addressed these limitations by controlling for both sex and age in our analyses. The stress group also showed significantly lower rates of follow-up compared to controls. However, there were no differences between those who remained in the study and those who were lost to follow-up on functional impairment at intake. In addition, the use of mixed regression analysis with random effects was used to mitigate this limitation. Finally, while the stress group was assessed within two months of parental diagnosis at baseline, on average, there was a wide range of time between diagnosis and the baseline assessment (range = 18–162 days). However, we accounted for days from diagnosis in our analyses.

Our finding of increased functional impairment in offspring of parents diagnosed with cancer is consistent with the literature on increased psychiatric symptoms in these offspring. We have previously reported that children who lose a parent early in life show functional impairment even 7 years after exposure to such childhood adversity [26]. Our study extends our prior findings to show that these offspring have higher scores of depression and anxiety symptoms, higher perceived stress, and worse functioning compared to controls as early as within two months of parental diagnosis. They also show worse functioning throughout the year following parental cancer diagnosis, an important outcome capturing the overall impact of stress in those with and without psychiatric disorders. Future studies with long-term follow-up are needed to track the course of psychiatric symptoms and functioning in offspring of parents diagnosed with cancer.

We also find early biological changes in these offspring with increased HCC within 2 months of parental diagnosis. In our study, the assessment of cortisol in hair permits an examination of mean levels since parental cancer diagnosis. We found that HCC decreased over time in the stress group and that higher salivary total diurnal cortisol was associated with increased functional impairment. This is the first study to examine HCC, along with additional biological measures of salivary cortisol and inflammation, in offspring of parents diagnosed with cancer within ~2 months of diagnosis, on average; furthermore, this study is the first to examine their relationships to overall functioning soon after stressor onset. Previous studies of adults exposed to trauma show reduced HCC [27] and economic disadvantage and maltreatment were found to be associated with reduced HCC in children [28,29]. A curvilinear relationship between HCC and depression symptoms was reported among adolescents, where both high and low HCC were associated with higher depressive symptoms [30]. Children and adolescents with PTSD symptoms were also found to have lower HCC compared to controls [31]. Other studies found no relationship between HCC and childhood psychopathology [32]. Similarly, both increased and attenuated salivary cortisol levels are reported in children in response to adversity including abuse, neglect, trauma, foster care, and parental loss and in relation to psychopathology [33,34,35,36,37,38]. These discrepant results could be due to methodological variations, differences in timing of assessment in relation to the stressor, or more complex non-linear relationships in implicated physiological pathways. This highlights the importance of assessing stress responses early on following the onset of the stressor and capturing the unfolding of stress responses in prospective studies.

This is the first study to examine HCC early on following the onset of a major life stressor in youth. Results suggest early activation of the cortisol responses and decreased levels of HCC over time in the stress group. However, HCC levels at follow-up continued to be higher in the stress group compared to controls. These results are consistent with an adaptive HPA axis response to stress early on, highlighting the importance of considering the timing of assessment relative to stress exposure in order to understand changes in these biological pathways. It is not clear whether the decrease in HCC levels observed in the stress group signifies a return to baseline levels or suggests a downregulation of HPA axis activity in response to chronic stress. Future studies with follow-up over longer periods of time are needed to corroborate either hypothesis, as well as to examine the psychological consequences of different trajectories of HPA axis activity in this population.

We found no significant differences in circulating levels of the inflammatory marker CRP among the offspring of parents with cancer compared to controls shortly after diagnosis, although there was a small effect size (d = 0.20). Studies show increased inflammation in adults and children exposed to childhood adversity [39,40]. We also found no group differences longitudinally and no group by time interactions in our mixed regression models. In addition, there was a small effect size for the relationship between CRP and functional impairment (d = 0.21). It is not clear whether these findings are due to our study’s small sample size or whether increased inflammation early on could reflect an adaptive and transient response to stress. Furthermore, only 9.1% of offspring in the stress group and 7.7% of the controls showed clinically significant functional impairment (GAS < 70). Thus, only a subset of offspring exposed to chronic stress will experience maladaptive responses. Future longitudinal studies with larger sample sizes with longer follow-up are needed to identify predictors of risk and resilience among offspring of parents diagnosed with cancer.

In conclusion, parental cancer is associated with alterations in cortisol response; additionally, these observed response dysregulations are associated with increased functional impairment in offspring. This study sheds light on the early biological and psychological stress responses in offspring of parents diagnosed with cancer, which could put them at increased risk for functional impairment and psychopathology. Clinicians need to assess and monitor psychiatric symptoms in offspring of parents with cancer early on in order to prevent the onset and long-term course of psychiatric disorders and functional impairment. Future studies with larger sample sizes and longer longitudinal follow-ups are needed to examine whether these biological markers could serve as predictors of early onset psychopathology following major life stressors.

Implications and Contribution: Parental cancer is associated with early biological changes in stress response that are associated with functional impairment in adolescents and young adults. Clinicians need to assess and monitor psychiatric symptoms and functioning in offspring of parents diagnosed with cancer early on following parental cancer diagnosis.

## Figures and Tables

**Table 1:** Demographics, clinical characteristics, and biological measures within ~2 months of parental cancer diagnosis.

	Controls	Cancer	Test	df	*p*	Cohen’s d
	*n* = 26	*n* = 32				
**Demographics**						
Age, in years, Mean ± SD	17.0 ± 3.5	17.0 ± 3.3	t = 0.1	56	0.95	0.02
Socioeconomic Status (SES), Mean ± SD	39.2 ± 4.7	41.2 ± 3.8	t = −1.6	45	0.12	0.47
Sex, % Female (*n*)	34.6 (9)	78.1 (25)	χ^2^ = 11.2	1	*0.001*	0.98
Body Mass Index (BMI), Mean ± SD	24.2 ± 4.3	23.7 ± 6.6	t = 0.3	50	0.77	−0.08
**Prior Psychiatric Disorders and Stressors**						
Childhood Trauma, Mean ± SD	30.5 ± 6.6	31.3 ± 5.4	t = −0.5	46	0.64	0.14
Negative Life Experiences, Mean ± SD	5.9 ± 8.5	6.6 ± 4.9	t = −0.6	56	0.81	0.08
History of Psychiatric Disorders, % Yes (*n*)	26.9 (7)	31.2 (10)	χ^2^ = 0.8	1	0.38	0.24
**Psychiatric Symptoms at Intake**						
Functional Impairment, Mean ± SD	87.8 ± 7.9	82.2 ± 10.5	t = 2.1	48	*0.04*	−0.60
Depression Symptoms, Mean ± SD	6.2 ± 6.7	11.6 ± 8.2	t = 2.8	54	*0.01*	0.80
Anxiety Symptoms, Mean ± SD	13.1 ± 12.5	23.7 ± 15.6	t = 2.5	50	*0.02*	0.73
PTSD Symptoms (PSS-I), Mean ± SD	2.0 ± 2.9	3.4 ± 3.8	t = 1.0	33	0.32	0.39
Sleep, Mean ± SD	3.6 ± 3.3	4.4 ± 3.0	t = 0.9	53	0.33	0.26
Perceived Stress Scale, Mean ± SD	16.8 ± 8.6	25.3 ± 9.9	t = 3.4	53	*0.003*	0.95
**Biological Measures at Intake**						
C-Reactive Protein (ln), Mean ± SD	−3.7 ± 1.9	−3.4 ± 2.1	t = 0.7	50	0.48	0.20
Total Diurnal Cortisol, AUC_cort_ * (ln), Mean ± SD	13.3 ± 0.8	13.2 ± 0.9	t = −0.04	41	0.94	−0.01
Cortisol Awakening Response, CAR (/1000), Mean ± SD	−0.3 ± 0.9	0.3 ± 1.2	t = −1.7	38	0.10	−0.53
Hair Cortisol Concentrations (HCC), Mean ± SD	5.8 ± 2.3	15.0 ± 8.5	t = 5.0	44	<*0.001*	2.21
Glucocorticoid Receptor or GR Sensitivity (AUC, /1000) ± SD	124.3 ± 81.6	121.0 ± 75.3	t = 0.2	48	0.88	−0.04
Stimulated IL-6 Production (/1000) ± SD	40.4 ± 22.5	34.2 ± 21.4	t = 1.0	48	0.32	−0.23

* Area under the curve for total cortisol output. Italics represent statistically significant results.

**Table 2:** Linear regression models for psychiatric symptoms and biological measures at intake controlling for covariates.

	Group (Cancer vs. Controls)			
	β	95% CI	*p*	d
**Psychiatric Symptoms**				
Functional Impairment (CGAS)	−5.5	−11.7, 0.7	0.08	−0.33
Depression Symptoms (CDI)	5.7	1.4, 9.9	*0.01*	0.50
Anxiety Symptoms (SCARED)	7.8	−2.6, 18.2	0.14	0.3
Perceived Stress Scale (PSS)	7.8	1.4, 14.3	*0.02*	0.48
**Biological Measures**				
CRP (ln)	0.4	−0.7, 1.5	0.46	0.19
AUC_cort_ (ln)	−0.1	−0.8, 0.6	0.78	−0.09
CAR	0.4	−0.7, 1.5	0.46	0.21
HCC	9.2	4.3, 14.1	*0.001*	1.47
GR Sensitivity	−1.5	−6.8, 3.7	0.57	−0.17
Stimulated Production of IL-6	−9.3	−22.1, 3.5	0.15	−0.43

Italics represent statistically significant results.

**Table 3:** Mixed regression models examining changes in functional impairment and biological measures over time.

	Model 1				Model 2			
	β	95% CI	*p*	d	β	95% CI	*p*	d
**Functional Impairment**								
Group *	−5.5	−10.4, −0.6	*0.03*	−0.40	−4.7	−9.8, 0.4	*0.07*	−0.31
Time	−0.001	−0.01, 0.01	0.92	−0.02	−0.001	−0.01, 0.01	0.90	0.01
Group X Time	0.005	−0.01, 0.02	0.60	0.09	0.005	−0.01, 0.02	0.56	0.07
Sex **	—	—	—	—	−1.9	−6.3, 2.5	0.40	−0.11
Age	—	—	—	—	−0.6	−1.1, 0.1	*0.09*	−0.30
**C-Reactive Protein (ln)**								
Group *	0.6	−0.5, 1.7	0.27	0.24	0.52	−0.5, 1.6	0.34	0.21
Time	0.001	−0.002, 0.003	0.66	0.09	0.001	−0.002, 0.003	0.80	0.06
Group X Time	−0.001	−0.01, 0.002	0.45	−0.16	−0.001	−0.01, 0.002	0.45	−0.16
Sex **	—	—	—	—	0.1	−0.9, 1.2	0.78	0.06
Age	—	—	—	—	0.07	−0.1, 0.2	0.29	0.23
BMI	—	—	—	—	0.2	0.08, 0.2	*<0.001*	0.93
**Total Diurnal Cortisol (AUC_cort_** **^ƒ^, ln)**								
Group *	−0.01	−0.5, 0.5	0.98	−0.01	−0.07	−0.6, 0.5	0.80	−0.06
Time	−0.001	−0.003, 0.002	0.65	−0.11	−0.001	−0.003, 0.002	0.51	−0.17
Group X Time	−0.001	−0.004, 0.002	0.71	−0.09	−0.001	−0.004, 0.003	0.74	−0.08
Sex **	—	—	—	—	0.4	−0.04, 0.9	0.07	0.46
Age	—	—	—	—	−0.001	−0.06, 0.06	0.97	−0.01
BMI	—	—	—	—	0.04	−0.01, 0.1	0.12	0.40
**Cortisol Awakening Response**								
Group *	0.4	−0.3, 1.1	0.26	0.30	0.1	−0.6, 0.8	0.70	0.10
Time	0.001	−0.002, 0.003	0.48	0.18	0.001	−0.001, 0.004	0.29	0.29
Group X Time	0.001	−0.002, 0.005	0.45	0.20	0.001	−0.002, 0.005	0.36	0.25
Sex **	—	—	—	—	0.6	−0.1, 1.3	0.10	0.45
Age	—	—	—	—	−0.09	−0.2, −0.004	*0.04*	0.58
BMI	—	—	—	—	0.01	−0.07, 0.09	0.08	0.08
**Hair Cortisol Concentrations**								
Group *	10.8	7.1, 14.4	*<0.001*	1.62	10.5	6.6, 14.5	*<0.001*	1.43
Time	−0.001	−0.01, 0.01	0.81	−0.05	−0.001	−0.01, 0.01	0.79	−0.06
Group X Time	−0.03	−0.04, −0.01	*<0.001*	−1.14	−0.03	−0.04, −0.01	*<0.001*	−1.08
Sex **	—	—	—	—	1.0	−2.2, 4.2	0.54	0.14
Age	—	—	—	—	0.3	−0.1, 0.8	0.14	0.33
BMI	—	—	—	—	0.06	−0.2, 0.3	0.66	0.10
GR Sensitivity *								
Group	4.8	−4.2, 5.1	0.84	0.05	−1.7	−4.9, 4.5	0.94	−0.02
Time	−0.03	−0.2, 0.2	0.72	−0.08	−0.1	−0.2, 0.1	0.61	−0.12
Group X Time	−0.1	−0.3, 0.2	0.69	−0.09	−0.02	−0.3, 0.2	0.86	−0.04
Sex **	—	—	—	—	−6.1	−4.2, 3.0	0.74	−0.08
Age	—	—	—	—	4.4	−0.5, 9.4	0.08	0.41
BMI	—	—	—	—	0.05	−3.2, 3.3	0.98	0.01
Stimulated Production of IL-6 *								
Group	−4.5	−1.7, 0.8	0.48	−0.16	−7.0	−1.9, 0.5	0.26	−0.26
Time	0.01	−0.05, 0.06	0.73	0.08	−0.01	−0.07, 0.04	0.61	−0.12
Group X Time	−0.03	−0.1, 0.04	0.43	−0.18	−0.002	−0.07, 0.07	0.94	−0.02
Sex **	—	—	—	—	2.5	−8.3, 13.3	0.65	0.10
Age	—	—	—	—	1.6	0.2, 3.0	*0.02*	0.54
BMI	—	—	—	—	−0.1	−1.0, 0.8	0.78	−0.06

* Cancer vs. control; ** males vs. female; **^ƒ^** area under the curve for total cortisol output. Italics represent statistically significant results.

**Table 4:** Relationship between functional impairment and biological variables.

	Functional Impairment			
	β	95% CI	*p*	d
**C-Reactive Protein**				
CRP (ln)	0.6	−0.4, 1.6	0.27	0.24
Group *	−4.8	−10.3, 0.6	0.08	−0.38
Time	−0.01	−0.02, 0.01	0.29	−0.23
Group X Time	−0.001	−0.02, 0.02	0.92	−0.02
**Total Diurnal Cortisol**				
AUC_cort_ ** (ln)	−3.0	−5.5, −0.5	*0.02*	−0.60
Group *	−5.9	−11.6, −0.1	*0.04*	−0.51
Time	−0.002	−0.02, 0.02	0.80	−0.06
Group X Time	−0.003	−0.03, 0.02	0.79	0.07
**Cortisol Awakening Response**				
CAR	−0.4	−2.0, 1.3	0.64	−0.12
Group *	−5.0	−11.5, 1.5	0.13	−0.40
Time	0.001	−0.02, 0.02	0.89	0.04
Group X Time	−0.004	−0.03, 0.02	0.73	−0.09
**Hair Cortisol Concentrations**				
HCC	−0.3	−0.6, 0.1	0.16	−0.31
Group *	−3.0	−9.1, 3.1	0.34	−0.21
Time	−0.01	−0.03, 0.01	0.31	−0.22
Group X Time	−0.002	−0.03, 0.02	0.87	0.03
**GR Sensitivity**				
GR Sensitivity	−0.003	−0.03, 0.02	0.82	−0.05
Group *	−3.6	−9.5, 2.3	0.23	−0.26
Time	−0.003	−0.02, 0.02	0.77	−0.06
Group X Time	−0.01	−0.03, 0.02	0.60	−0.11
**Stimulated Production of IL-6**				
Stimulated Production of IL-6	−0.001	−0.08, 0.08	0.98	0.01
Group *	−3.6	−9.5, 2.3	0.23	−0.27
Time	−0.003	−0.02, 0.02	0.79	−0.06
Group X Time	−0.01	−0.03, 0.02	0.60	−0.12

* Cancer vs. control; ** area under the curve for total cortisol output. Italics represent statistically significant results.

## Data Availability

The data presented in this study are available on request from the corresponding author.

## References

[1] Mariotto A.B., Robin Yabroff K., Shao Y., Feuer E.J., Brown M.L. (2011). Projections of the cost of cancer care in the United States: 2010–2020. J. Natl. Cancer Inst..

[2] Bradley C.J., Yabroff K.R., Dahman B., Feuer E.J., Mariotto A., Brown M.L. (2008). Productivity costs of cancer mortality in the United States: 2000–2020. J. Natl. Cancer Inst..

[3] Weaver K.E., Rowland J.H., Alfano C.M., McNeel T.S. (2010). Parental cancer and the family. Cancer.

[4] Morris J.N., Martini A., Preen D. (2016). The well-being of children impacted by a parent with cancer: An integrative review. Supportive Care Cancer.

[5] Brent D.A., Melhem N.M., Masten A.S., Porta G., Payne M.W. (2012). Longitudinal Effects of Parental Bereavement on Adolescent Developmental Competence. J. Clin. Child Adolesc. Psychol..

[6] Melhem N.M., Porta G., Shamseddeen W., Payne M.W., Brent D.A. (2011). Grief in children and adolescents bereaved by sudden parental death. Arch. Gen. Psychiatry.

[7] Bylund-Grenklo T., Fürst C.J., Nyberg T., Steineck G., Kreicbergs U. (2016). Unresolved grief and its consequences. A nationwide follow-up of teenage loss of a parent to cancer 6–9 years earlier. Supportive Care Cancer.

[8] Brent D., Melhem N., Donohoe M.B., Walker M. (2009). The Incidence and Course of Depression in Bereaved Youth 21 Months after the Loss of a Parent to Suicide, Accident, or Sudden Natural Death. Am. J. Psychiatry.

[9] Miller G.E., Chen E., Parker K.J. (2011). Psychological stress in childhood and susceptibility to the chronic diseases of aging: Moving toward a model of behavioral and biological mechanisms. Psychol. Bull..

[10] Slavich G.M., Irwin M.R. (2014). From Stress to Inflammation and Major Depressive Disorder: A Social Signal Transduction Theory of Depression. Psychol. Bull..

[11] Danese A., Pariante C.M., Caspi A., Taylor A., Poulton R. (2007). Childhood maltreatment predicts adult inflammation in a life-course study. Proc. Natl. Acad. Sci. USA.

[12] Andreasen N.C. (1977). The Family History Method Using Diagnostic Criteria. Arch. Gen. Psychiatry.

[13] Shaffer D. (1983). A Children’s Global Assessment Scale (CGAS). Arch. Gen. Psychiatry.

[14] Cohen S., Kamarck T., Mermelstein R. (1983). A Global Measure of Perceived Stress. J. Health Soc. Behav..

[15] Birmaher B., Khetarpal S., Brent D., Cully M., Balach L., Kaufman J., Neer S.M. (1997). The Screen for Child Anxiety Related Emotional Disorders (SCARED): Scale construction and psychometric characteristics. J. Am. Acad. Child Adolesc. Psychiatry.

[16] Kovacs M. (1985). The Children’s Depression, Inventory (CDI). Psychopharmacol. Bull..

[17] Foa E.B., Riggs D.S., Dancu C.V., Rothbaum B.O. (1993). Reliability and validity of a brief instrument for assessing post-traumatic stress disorder. J. Trauma. Stress.

[18] Pennebaker J.W., Susman J.R. (1988). Disclosure of traumas and psychosomatic processes. Soc. Sci. Med..

[19] Gray M.J., Litz B.T., Hsu J.L., Lombardo T.W. (2004). Psychometric properties of the life events checklist. Assessment.

[20] Buysse D.J., Reynolds C.F., Monk T.H., Berman S.R., Kupfer D.J. (1989). The Pittsburgh Sleep Quality Index: A new instrument for psychiatric practice and research. Psychiatry Res..

[21] Hollingshead A.A. (1975). Four-Factor Index of Social Status.

[22] Stalder T., Kirschbaum C. (2012). Analysis of cortisol in hair—State of the art and future directions. Brain Behav. Immun..

[23] Laudenslager M.L., Jorgensen M.J., Grzywa R., Fairbanks L.A. (2011). A novelty seeking phenotype is related to chronic hypothalamic-pituitary-adrenal activity reflected by hair cortisol. Physiol. Behav..

[24] Pruessner J.C., Kirschbaum C., Meinlschmid G., Hellhammer D.H. (2003). Two formulas for computation of the area under the curve represent measures of total hormone concentration versus time-dependent change. Psychoneuroendocrinology.

[25] Collins L.M., Schafer J.L., Kam C.M. (2001). A comparison of inclusive and restrictive strategies in modern missing data procedures. Psychol. Methods.

[26] Pham S., Porta G., Biernesser C., Walker Payne M., Iyengar S., Melhem N., Brent D.A. (2018). The burden of bereavement: Early-onset depression and impairment in youths bereaved by sudden parental death in a 7-year prospective study. Am. J. Psychiatry.

[27] Steudte S., Kirschbaum C., Gao W., Alexander N., Schönfeld S., Hoyer J., Stalder T. (2013). Hair cortisol as a biomarker of traumatization in healthy individuals and posttraumatic stress disorder patients. Biol. Psychiatry.

[28] Gray N.A., Dhana A., Van Der Vyver L., Van Wyk J., Khumalo N.P., Stein D.J. (2018). Determinants of hair cortisol concentration in children: A systematic review. Psychoneuroendocrinology.

[29] White L.O., Ising M., von Klitzing K., Sierau S., Michel A., Klein A.M., Andreas A., Keil J., Quintero L., Müller-Myhsok B. (2017). Reduced hair cortisol after maltreatment mediates externalizing symptoms in middle childhood and adolescence. J. Child Psychol. Psychiatry.

[30] Ford J.L., Boch S.J., Browning C.R. (2019). Hair cortisol and depressive symptoms in youth: An investigation of curvilinear relationships. Psychoneuroendocrinology.

[31] Straub J., Klaubert L.M., Schmiedgen S., Kirschbaum C., Goldbeck L. (2017). Hair cortisol in relation to acute and post-traumatic stress symptoms in children and adolescents. Anxiety Stress Coping.

[32] Fuchs A., Jaite C., Neukel C., Dittrich K., Bertsch K., Kluczniok D., Möhler E., Attar C.H., Brunner R., Bödeker K. (2018). Link between children’s hair cortisol and psychopathology or quality of life moderated by childhood adversity risk. Psychoneuroendocrinology.

[33] Bick J., Nguyen V., Leng L., Piecychna M., Crowley M.J., Bucala R., Mayes L.C., Grigorenko E.L. (2015). Preliminary associations between childhood neglect, MIF, and cortisol: Potential pathways to long-term disease risk. Dev. Psychobiol..

[34] Dietz L.J., Stoyak S., Melhem N., Porta G., Matthews K.A., Payne M.W., Brent D.A. (2013). Cortisol response to social stress in parentally bereaved youth. Biol. Psychiatry.

[35] McLaughlin K.A., Sheridan M.A., Tibu F., Fox N.A., Zeanah C.H., Nelson C.A. (2015). Causal effects of the early caregiving environment on development of stress response systems in children. Proc. Natl. Acad. Sci. USA.

[36] Morris M.C., Compas B.E., Garber J. (2012). Relations among posttraumatic stress disorder, comorbid major depression, and HPA function: A systematic review and meta-analysis. Clin. Psychol. Rev..

[37] Pfeffer C.R., Altemus M., Heo M., Jiang H. (2007). Salivary Cortisol and Psychopathology in Children Bereaved by the September 11, 2001 Terror Attacks. Biol. Psychiatry.

[38] Stetler C., Miller G.E. (2011). Depression and Hypothalamic-Pituitary-Adrenal Activation: A Quantitative Summary of Four Decades of Research. Psychosom. Med..

[39] Copeland W.E., Shanahan L., Worthman C., Angold A., Costello E.J. (2012). Cumulative Depression Episodes Predict Later C-Reactive Protein Levels: A Prospective Analysis. Biol. Psychiatry.

[40] Miller G.E., Cole S.W. (2012). Clustering of depression and inflammation in adolescents previously exposed to childhood adversity. Biol. Psychiatry.

